# *Mycobacterium tuberculosis* carrying the rifampicin drug-resistance-conferring *rpoB* mutation H445Y is associated with suppressed immunity through type I interferons

**DOI:** 10.1128/mbio.00946-23

**Published:** 2023-09-08

**Authors:** Suhas Bobba, Nicole C. Howard, Shibali Das, Mushtaq Ahmed, Linrui Tang, Shyamala Thirunavukkarasu, Michelle H. Larsen, Barun Mathema, Maziar Divangahi, Shabaana A. Khader

**Affiliations:** 1 Department of Molecular Microbiology, Washington University School of Medicine, St. Louis, Missouri, USA; 2 Department of Microbiology, University of Chicago, Chicago, Illinois, USA; 3 Department of Epidemiology, Columbia University Mailman School of Public Health, New York, New York, USA; 4 Department of Microbiology and Immunology, Albert Einstein College of Medicine, Bronx, New York, USA; 5 Meakins-Christie Laboratories, Department of Medicine, McGill University, Montreal, Quebec, Canada; 6 Department of Microbiology and Immunology, McGill International TB Centre, Montreal, Quebec, Canada; 7 Department of Pathology, McGill University Health Centre, Montreal, Quebec, Canada; National Institute of Allergy and Infectious Diseases, Bethesda, Maryland, USA; University of California, San Francisco, San Francisco, California, USA

**Keywords:** *Mycobacterium tuberculosis*, type I interferons, inflammation, drug-resistant mutations, lung infection

## Abstract

Tuberculosis (TB) is one of the leading causes of death due to an infectious disease. The rise of multi-drug resistance (MDR) in *Mycobacterium tuberculosis* (*Mtb*), the causative agent of TB, presents a significant obstacle to TB control. While human studies report dysregulated immune responses during MDR TB, a clear understanding of the host-pathogen interactions of MDR *Mtb* is lacking. Here, we studied the immune responses induced by *Mtb* strains carrying two of the most common rifampicin drug-resistance (RDR)-conferring single-nucleotide polymorphisms (SNPs) in the RNA polymerase gene of *Mtb*, which accounts for nearly 90% of drug-resistance mutations found clinically in *Mtb*. During *Mtb* infection of primary human macrophages, we found that pro-inflammatory cytokine production was reduced upon infection with *Mtb* carrying the H445Y SNP but not the S450L SNP. Using a mouse model, we also characterized the host immune response *in vivo* following infection. Despite similar establishment of *Mtb* infection in the lung and dissemination to the peripheral organs, we show that infection with the RDR *Mtb rpoB*-H445Y strain, but not with the *rpoB*-S450L strain, resulted in a suppressed lung myeloid and lymphoid immune responses through type I IFN-dependent pathways, relative to *wt Mtb*. This suppressed host immunity had functional consequences in limiting control of RDR *Mtb* strains harboring *rpoB*-H445Y and led to worse pathology during chronic TB disease. Collectively, our results suggest that disease pathogenesis may be associated with specific RDR mutations in *Mtb*, which may differentially regulate immune responses.

**IMPORTANCE:**

This study highlights the impact of specific rifampicin-resistance-conferring mutations on the host immune response to *Mycobacterium tuberculosis* (*Mtb*), the causative agent of tuberculosis (TB). Clinical reports have previously suggested that multi-drug-resistant) TB patients exhibit altered peripheral immune responses as compared with their drug-sensitive TB counterparts. The murine model of infection with *Mtb* strains carrying drug-resistance-conferring mutations recapitulated these findings and allowed us to mechanistically interrogate the pathways responsible for driving the divergent immune responses. Our findings underscore the need for greater investigation into bacterial heterogeneity to better appreciate the diversity in host-pathogen interactions during TB disease.

## INTRODUCTION

Tuberculosis (TB) is a major global health threat, with over 10 million people estimated to have developed active disease in 2021 (1). Emergence of *Mycobacterium tuberculosis* (*Mtb*) strains resistant to the frontline antibiotic, rifampicin, has significantly limited global efforts to control TB. In 2021 alone, nearly 600,000 TB cases were found to be rifampicin drug resistant (RDR), which requires expanded use of more toxic second-line antibiotics. A bottleneck to controlling RDR *Mtb* infection is our lack of clear understanding of the disease pathogenesis following DR *Mtb* infection. The immune responses and clinical outcomes in individuals infected with DR *Mtb* can differ when compared with TB patients infected with drug-susceptible (DS) *Mtb*. In particular, while there is evidence of impaired CD4^+^ T helper type 1 (Th1) responses and dysregulation of the Th1/T helper type 2 (Th2) balance in humans with multi-drug-resistant (MDR) TB (2 – 5), the underlying mechanisms that contribute to this impairment are not known.

The two most common DR mutations that confer resistance to rifampicin are H445Y and S450L in the RNA polymerase (*rpoB*) subunit of *Mtb,* which account for nearly 90% of drug-resistance mutations found clinically in Mtb (6 – 9). We recently showed that MDR *Mtb* harboring the mutation *rpoB*-H445Y, but not *rpoB*-S450L, differentially expressed *Mtb* surface lipids and induced macrophage reprogramming via a type I interferon (IFN) response in murine macrophages *in vitro* (10). In the current study, we investigated how drug-resistance-conferring mutations may impact the immune response following DR *Mtb* infection *in vivo*. We found that *Mtb* harboring the *rpoB-*H445Y SNP drove reduced pro-inflammatory cytokine production, namely IL-1, in infected human macrophages. Using a mouse model, we showed that *Mtb* infection with a strain carrying the H445Y SNP, but not the S450L SNP, resulted in impaired recruitment of myeloid and lymphoid cells *in vivo*. Importantly, we show that the suppressed immune response in the lung associated with infection with the *rpoB*-H445Y strain is dependent on type I IFN signaling mechanisms. Thus, our findings highlight how RDR mutations in *Mtb* may trigger type I IFNs to suppress immune responses and drive divergent immune responses and pathology when compared with DS *Mtb*. Our results contribute to our understanding of immune responses to major RDR *Mtb* infections and provide models and new avenues to mechanistically interrogate immunomodulation in DR and MDR TB patients.

## RESULTS

### The presence of drug-resistant rpoB-H445Y mutation drives differential pro-inflammatory cytokine production in *Mtb*-infected murine and human macrophages

We have previously reported that multi-drug-resistant clinical isolates, such as W_7642, harboring the specific drug-resistance-conferring mutation, H445Y, but not S450L in the RNA polymerase (*rpoB*) subunit of *Mtb*, modulate *Mtb* interactions with murine macrophages and drive differential cytokine production in macrophages (10), especially limiting interleukin-1 (IL-1) and increasing IFN production. We generated a collection of independently isolated drug-resistant mutants through plating on selective media containing rifampicin (Fig. S1). Comparative whole-genome sequence analysis between these *Mtb* mutants with the parental *wt* HN878 *Mtb* strain revealed various non-overlapping non-synonymous single-nucleotide polymorphisms (nsSNPs) (Fig. S1A). We observed that many of these nsSNPs, such as those in *pks4* and *pks12*, are commonly occurring in independent isolates across the different *rpoB* mutants. When we infected bone marrow-derived macrophages (BMDMs) from C57Bl/6 (B6) mice, only the *Mtb* strains containing the *rpoB*-H445Y mutation, and a mutation in a probable transcriptional regulatory protein *Rv0465c,* significantly induced IFN production upon infection (Fig. S1B). Additionally, a comparative analysis of the MDR clinical isolate that we previously characterized (W_7642) (10) with three strains, HN878, HN13 (*rpoB-*S450L), and HN14 (*rpoB-*H445Y), revealed one shared nsSNP in *rpoB* that was present only in the *rpoB-*H445Y strain (Table 1). Despite the absence of isogenic strains due to technical limitations, these findings bolstered our interpretation of the role of a specific mutation in *rpoB* in altering host responses during *Mtb* infections.

**TABLE 1:** Comparison of SNPs across spontaneous mutants^*a*^

Pos	*Wt*	*rpoB*-S450L	*rpoB*-H445Y	AA_POS	Effect	Locus_tag	Gene	Product
555619	T	T	C	230/474	Missense_variant c.688A > G p.Met230Val	Rv0465c		Probably transcriptional regulatory protein
**761139**	**C**	**C**	**T**	**445/1172**	**Missense_variant c.1333C > T p.His445Tyr**	**Rv0667**	***rpoB***	**DNA-directed RNA polymerase (beta chain) RpoB (transcriptase beta chain) (RNA polymerase beta subunit**)
761155	C	T	C	450/1172	Missense_variant c.1349C > T p.Ser450Leu	Rv0667	*rpoB*	DNA-directed RNA polymerase (beta chain) RpoB (transcriptase beta chain) (RNA polymerase beta subunit)
972980	C	T	C	243/386	Missense_variant c.727G > A p.Gly243Ser	Rv0874c		Conserved hypothetical protein
1155197	A	A	G	492/709	Missense_variant c.1474A > G p.Ile492Val	Rv1030	*kdpB*	Probable potassium-transporting P-type ATPase B chain KdpB
1319222	C	C	T	1330/1582	Missense_variant c.3989C > T p.Ala1330Val	Rv1181	*pks4*	Probably polyketide beta-ketoacyl synthase Pks4
2300546	A	T	A	2147/4151	Missense_variant c.6441T > A p.His2147Gln	Rv2048c	*pks12*	Polyketide synthase Pks12
3690948	C	C	T					
3804941	G	G	A	26/236	Missense_variant c.77G > A p.Cys26Tyr	Rv3390	*lpqD*	Probable conserved lipoprotein LpqD
3846791	A	G	A					
3846851	A	G	A					
3846852	G	C	G					
3846853	C	T	C					
3846857	A	G	A					
3846860	G	T	G					
3846866	A	C	A					

^*a*^ Based on the whole-genome sequencing, all unique non-synonymous SNPs, including the intergenic ones, for the *rpoB*-S450L and *rpoB*-H445Y strains were determined in reference to the *wt* parental *Mtb* strain HN878. The only SNP shared by any of these strains and the previously characterized W_7642 is boldfaced and is present in the *rpoB*-H445Y strain.

**Fig 1:**
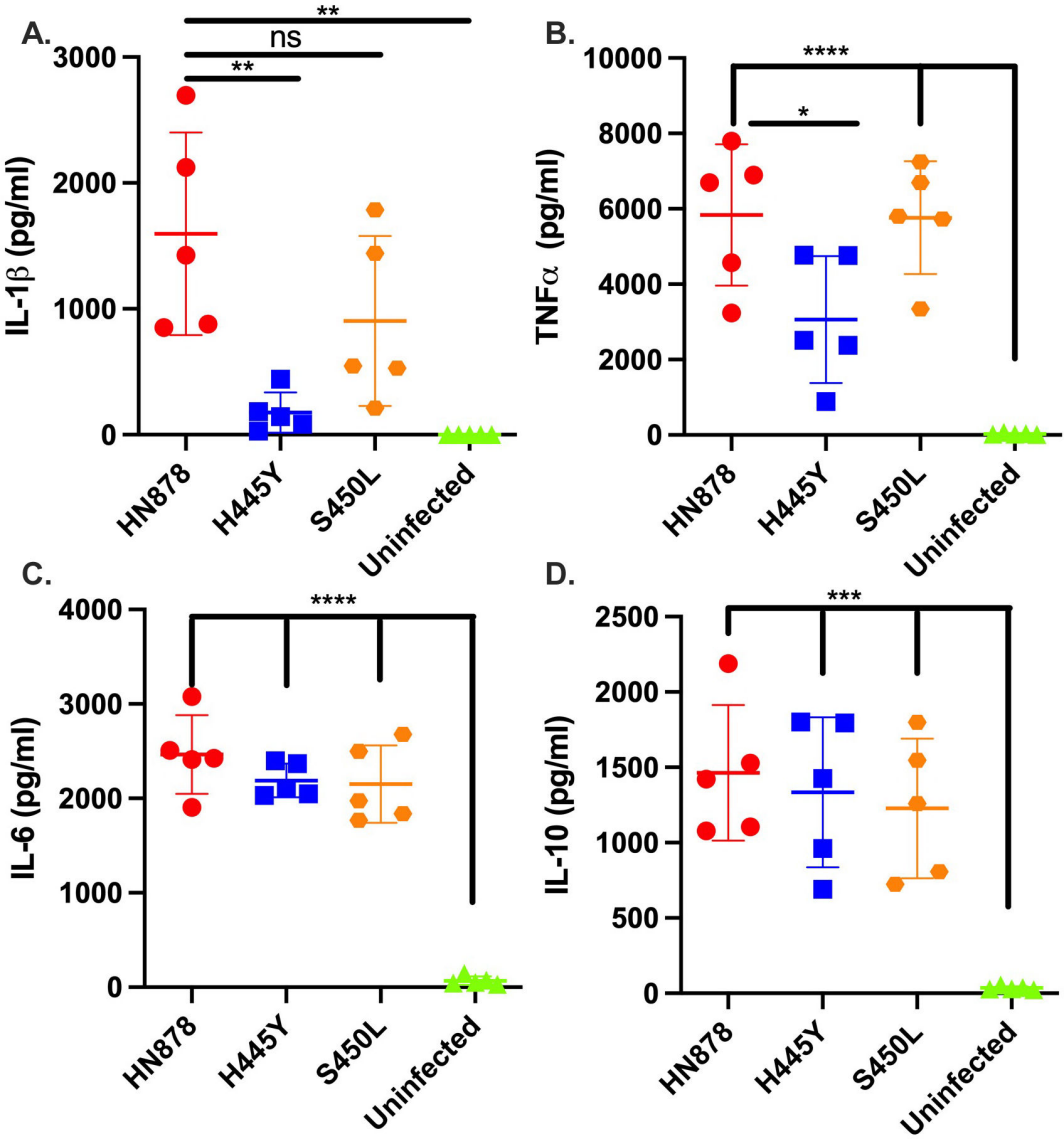
*Mycobacterium tuberculosis strains* with different *rpoB* SNPs drive divergent cytokine responses in infected human monocyte-derived macrophages. CD14^+^ monocytes that were isolated from the peripheral blood of five different donors and differentiated into macrophages were infected with *wt* (HN878), *rpoB*-H445Y, or *rpoB*-S450L *Mtb* strains at an MOI of 1. After 6 days post infection (dpi), supernatants were collected. (**A–D**) Cytokine protein levels in the supernatants were measured by Lincoplex. Each data point shown represents the average of three technical replicates from an individual donor. All data sets were tested for normality using the Shapiro-Wilk and passed (*P* > 0.05). Significant differences are indicated with asterisks (*P < 0.05, **P < 0.01, ***P < 0.001, ****P < 0.0001) by ordinary one-way ANOVA with Tukey’s post-tests (**A–D**).

To extend the clinical relevance of our findings, we next studied the impact of either H445Y or S450L DR SNPs on cytokine responses following infection of human monocyte-derived macrophages (MDMs). We found that both DS *wt Mtb* (HN878) and the DR S450L mutant *Mtb* (HN13 *rpoB-*S450L) induced IL-1 production upon infection (Fig. 1A), while H445Y mutant *Mtb* (HN14 *rpoB-*H445Y) infection did not induce increased IL-1 production. These findings are in concert with our previously published findings, where the clinical W_7642 MDR *Mtb* strain also induced limited IL-1 production in murine macrophages upon infection (10). Additionally, infection with HN878 and *rpoB-*S450L *Mtb* also resulted in increased TNF production, while *rpoB-*H445Y infection did not (Fig. 1B). Infection with all three strains (HN878, *rpoB-*S450L, and *rpoB-*H445Y) resulted in increased production of other cytokines that were measured, such as the pro-inflammatory IL-6 and the anti-inflammatory IL-10 (Fig. 1C and D). Levels of IFN were below the level of detection. With our previously published findings (10), our data together show that the RDR *Mtb* carrying H445Y SNP and the S450L SNP elicit highly divergent host macrophage responses, with the *rpoB-*H445Y *Mtb* strain limiting the production of pro-inflammatory cytokines in infected human macrophages.

### Infection with rpoB-H445Y *Mtb* results in limited lung immune responses in mice

**Fig 2:**
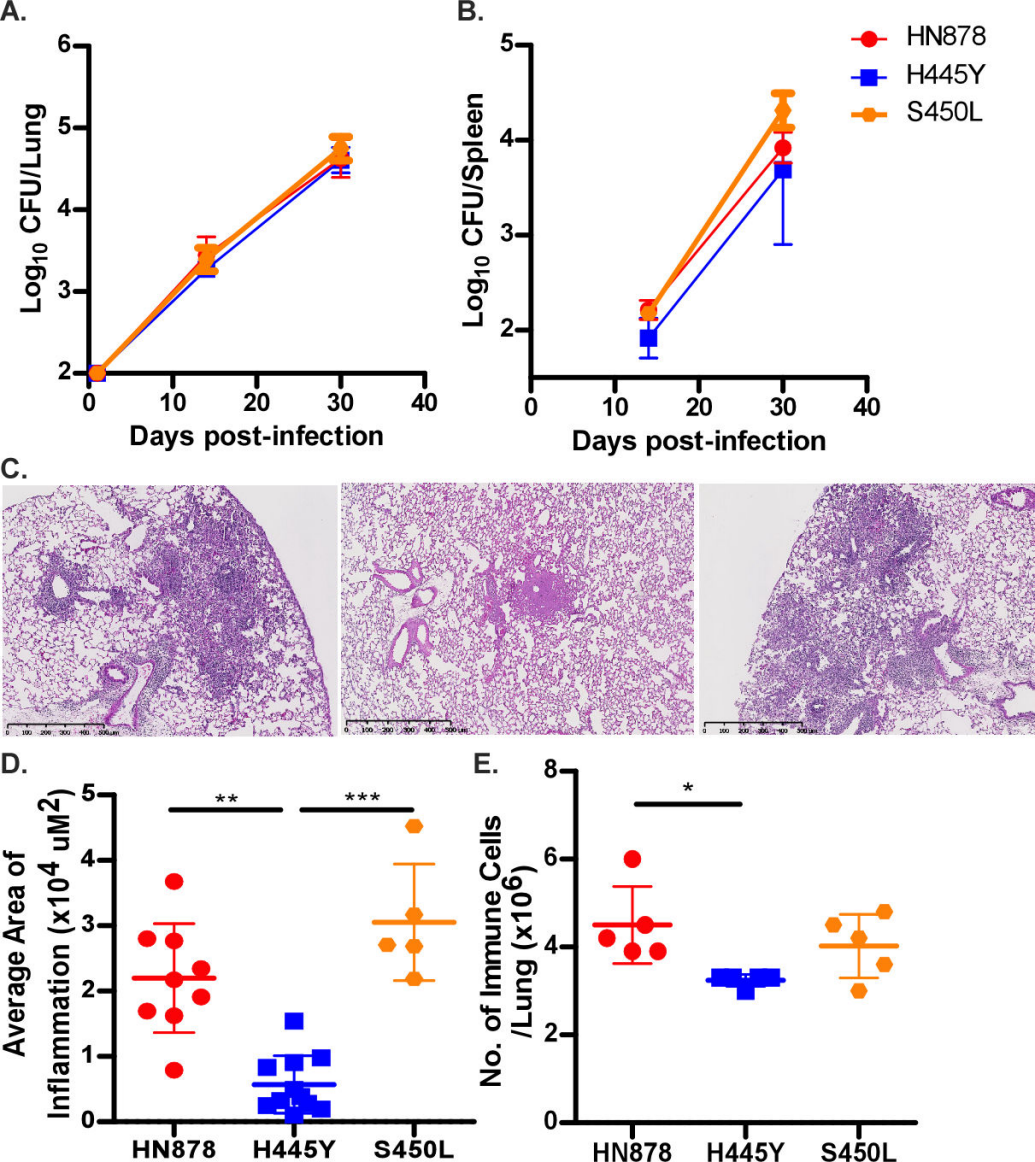
*Mtb* with different *rpoB* SNPs induce dissimilar pathology in infected mice. C57Bl/6 (B6) mice were infected with a low dose (~100 CFU) of *Wt HN878*, *rpoB*-H445Y, or *rpoB*-S450L *Mtb* by the aerosol route. Bacterial burden in (**A**) lung and (**B**) spleen was determined by plating at 14 and 30 dpi. (**C**) Formalin-fixed paraffin-embedded (FFPE) lung sections from mice infected for 30 days were hematoxylin and eosin (H&E) stained, and representative images for each group are shown: left (*Wt HN878*), middle (*rpoB*-H445Y), or right (*rpoB*-S450L *Mtb*). (**D**) The inflammatory area was measured. (**E**) Total numbers of lung immune cells in single-cell suspensions were determined. The data shown represent the means ± SD of 5–10 biological replicates per experiment. Significant differences are indicated with asterisks (*P < 0.05; **P < 0.01; ***P < 0.001) by Kruskal-Wallis test with Dunn’s multiple comparisons tests (**A–E**). One of three independent experiments is shown.

**Fig 3:**
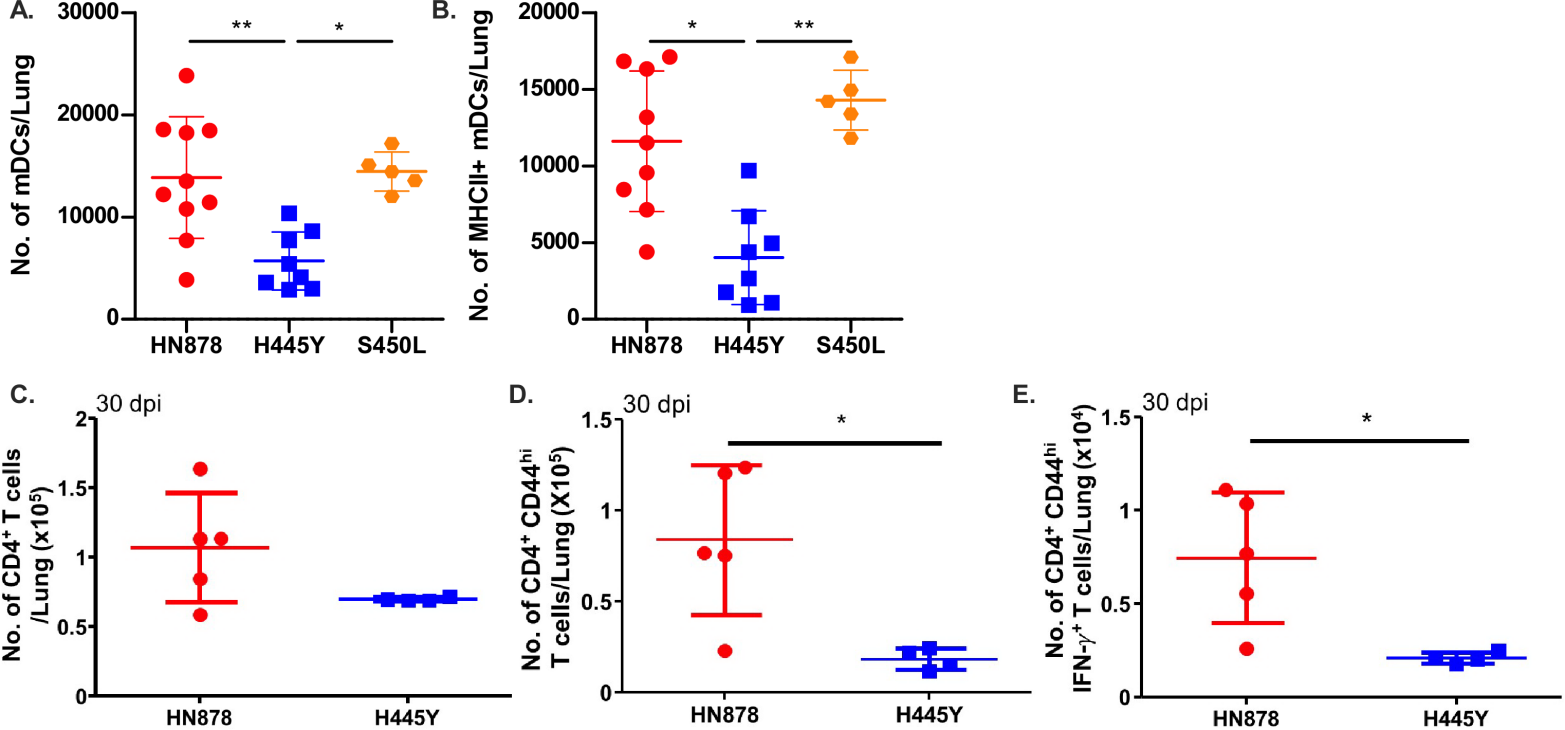
*rpoB*-H445Y *Mtb* infection induces limited myeloid dendritic cell accumulation and subsequent T cell responses. B6 mice were infected with a low dose (~100 CFU) of *Wt*, *rpoB*-H445Y, or *rpoB*-S450L *Mtb* by the aerosol route and sacrificed at 30 dpi for analysis. (**A**) Total number of mDCs and (**B**) MHC Class II^+^ mDCs were determined by flow cytometry. The numbers of (**C**) total, (**D**) CD44^hi^, and (**E**) IFN^+^ CD4^+^ T cells were determined by flow cytometry following *ex vivo* stimulation with purified *Mtb* antigens ESAT6 and Ag85B. The data shown represent the means ± SD of 4–10 biological replicates per experiment. Significant differences are indicated with asterisks (*P < 0.05; **P < 0.01) by Kruskal-Wallis test with Dunn’s multiple comparisons tests (**A–E**). One of three independent experiments is shown.

The impact of DR *Mtb* infection on cytokine responses likely impacts TB disease progression *in vivo*. Thus, C57BL/6J (B6) mice were infected with *Mtb* HN878, *rpoB-*H445Y, or *rpoB-*S450L (10 – 12). We found that infection with all *Mtb* strains established similar lung bacterial burden and disseminated effectively to the spleen at 14 and 30 dpi (Fig. 2A and B). Importantly, despite similar bacterial loads, *rpoB*-H445Y *Mtb* infection induced significantly lower pulmonary inflammation when compared to *wt Mtb* (HN878) and *rpoB*-S450L infections, with *rpoB*-H445Y *Mtb* infection inducing smaller-sized inflamed lesions (Fig. 2C and D). This coincided with reduced accumulation of lung immune cells after *rpoB*-H445Y *Mtb* infection, compared with *wt* HN878 *and* rpoB-S450L *Mtb* infections (Fig. 2E). Characterization of these immune populations revealed that the accumulation of lung myeloid dendritic cells (mDCs) was significantly reduced in *rpoB*-H445Y *Mtb*-infected mice when compared with either *wt* HN878 or *rpoB*-S450L *Mtb*-infected mice (Fig. 3A). Similar defects in the accumulation of lung neutrophils, recruited macrophages (RMs), and monocytes were also observed in *rpoB*-H445Y *Mtb-*infected mice when compared with *Wt Mtb* infection (Fig. S2A through C). Upregulation of Major Histocompatibility Complex (MHC) Class II expression on lung macrophages is associated with *Mtb* control (13). Fewer MHC Class II-expressing lung mDCs were also observed in *rpoB*-H445Y *Mtb* infection in contrast with either *wt* HN878 or *rpoB*-S450L *Mtb* infections (Fig. 3B). The numbers of other MHC Class II-expressing lung myeloid cells, RMs and monocytes, were also reduced in *rpoB*-H445Y *Mtb-*infected mouse lungs when compared to *Wt Mtb* infection (Fig. S2D and E). During *Mtb* infection, MHC Class II expression on mDCs is critical for antigen presentation to CD4^+^ T cells (13). Based on the fewer numbers of activated myeloid cells in *rpoB*-H445Y infection, we next examined T cell responses in the lungs of mice infected with *Wt Mtb* or *rpoB*-H445Y *Mtb*. While the total number of CD4^+^ T cells was similar in the lungs of mice infected with *Wt* or *rpoB*-H445Y *Mtb* (Fig. 3C), accumulation of activated CD4^+^CD44^hi^ T cells was significantly reduced in the lungs of *rpoB*-H445Y *Mtb*-infected mice (Fig. 3D). Indeed, in mice infected with the *rpoB-*H445Y *Mtb,* there were significantly fewer *Mtb*-specific activated lung CD4^+^ CD44^hi^ T cells, which produced IFN when compared with *Wt Mtb* infection (Fig. 3E). These results show that while *wt* and S450L SNP carrying *Mtb* drive similar activation of the immune response *in vivo*, *Mtb* harboring the H445Y SNP may be associated with a limited immune response in the lung in infection, characterized by reduced inflammation and decreased accumulation of activated myeloid and T cells.

Infection with *rpoB*-H445Y *Mtb* resulted in higher lung IFN but lower IL-1 levels, relative to *Wt Mtb* infection (Fig. S3A and B). We also found reduced expression of chemokines in *rpoB*-H445Y *Mtb*-infected lungs, including keratinocytes-derived chemokine (KC), a neutrophil chemo-attractant, MIP1, a monocyte/macrophage chemo-attractant, and regulated upon activation, normal T cell expressed and presumably secreted (RANTES), a T cell and myeloid cell chemoattractant, in *rpoB*-H445Y *Mtb* infection (Fig. S3C through E). Assessment of markers of proliferation and cell death in the lung myeloid cell subsets at this time point showed no differences between *Wt* and *rpoB*-H445Y *Mtb*-infected mice (data not shown). Thus, these data collectively indicate that the reduction in chemokines may be driving reduced immune cell recruitment, which underlies the limited accumulation of immune cells in *rpoB*-H445Y *Mtb* infection.

Using the susceptible TB mouse model, C3HeB/FeJ (FeJ) mice, which exhibit necrotic TB granulomas similar to human TB (14), infection with *rpoB*-H445Y *Mtb* also resulted in reduced pulmonary inflammation and also induced lower IL-1 levels, relative to *wt* HN878 *Mtb* infection (Fig. S4A and B). Similar to our findings in B6 mice, *rpoB*-H445Y *Mtb* infection resulted in reduced accumulation of mDCs and fewer MHC Class II-expressing mDCs (Fig. S4C and D). When compared with *Wt Mtb* infection, FeJ mice infected with *rpoB*-H445Y *Mtb* also had fewer numbers of IFN-producing, activated lung CD4^+^CD44^hi^ (Fig. S4E). These data suggest a likely association between the H445Y SNP and reduced lung myeloid and T cell recruitment and activation across resistant and susceptible mouse models of TB.

### Reduced lung T cell responses in rpoB-H445Y *Mtb* infection are associated with decreased trafficking of mDCs to lymph nodes

**Fig 4:**
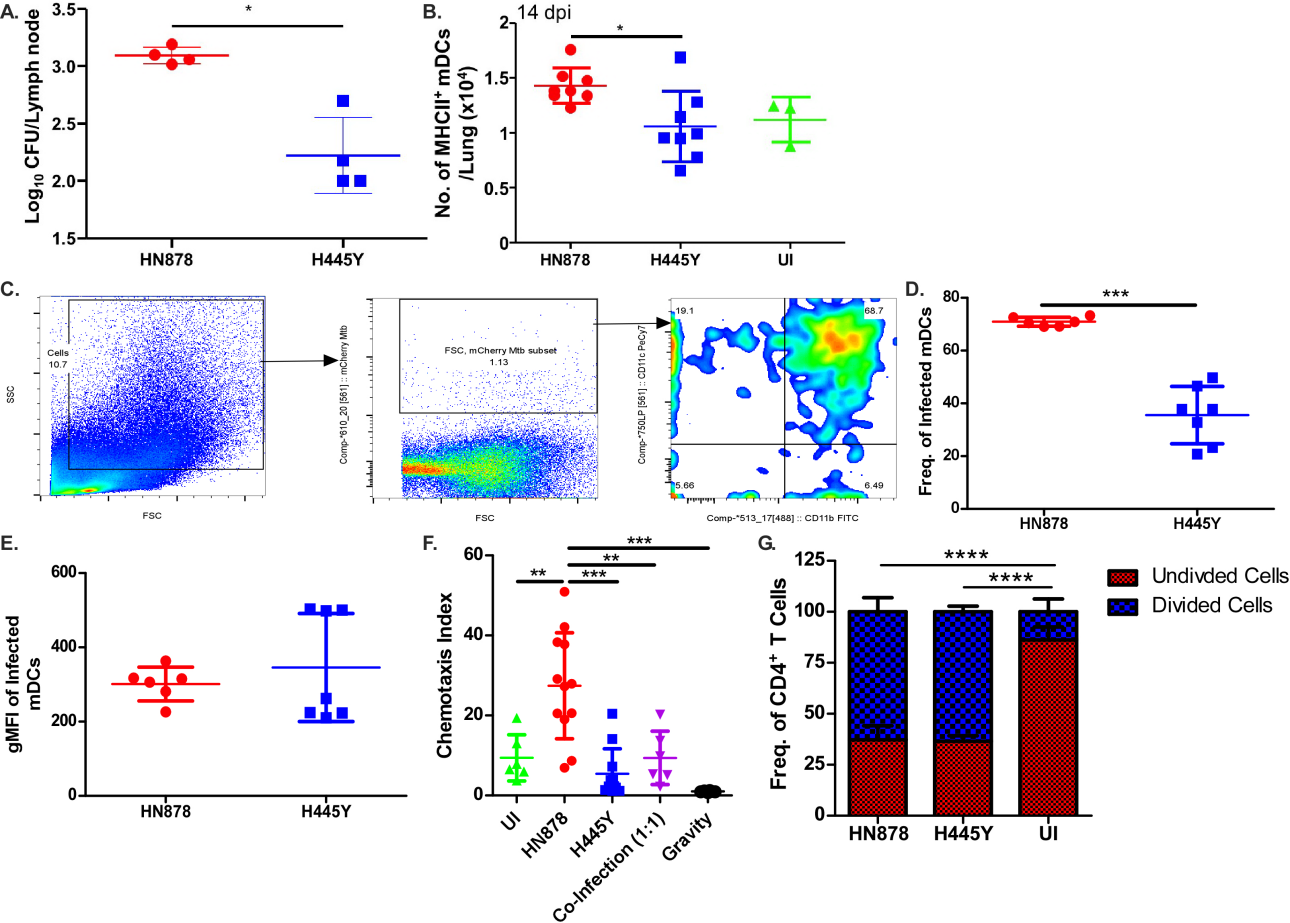
Impaired T cell responses are driven by defects in myeloid dendritic cell migration in *rpoB*-H445Y *Mtb* infection. B6 mice were aerosol infected with a low dose of either *Wt* or *rpoB*-H445Y *Mtb* and sacrificed at 14 dpi for analysis. (**A**) Bacterial burden in the lymph nodes was determined by plating. (**B**) Numbers of MHC Class II^+^ mDCs were determined by flow cytometry. Uninfected mice were also included (*n* = 3). B6 mice were aerosol infected with a low dose of either mCherry-expressing *Wt* or *rpoB*-H445Y *Mtb*. (**C**) Gating strategy for *Mtb*-infected cells in the lung is shown. (**D**) The frequencies of *Mtb*-infected lung cells that are mDCs (CD11c^+^ CD11b^+^) and (**E**) the geometric mean fluorescent intensity (MFI) of mCherry expression in infected mDCs were calculated. BMDCs were infected with either *Wt* or *rpoB*-H445Y *Mtb* or co-infected at a ratio of 1:1 *Wt:rpoB*-H445Y at a multiplicity of infection (MOI) of 1. (**F**) One day post infection, *in vitro* chemotaxis assays were carried out toward CCL-19, and a chemotaxis index was calculated. (**G**) One day post infection, BMDCs were co-cultured with isolated, enriched, and carboxyfluorescein succinimidyl ester (CFSE)-labeled CD4^+^ T cells from naïve ESAT-6 ab TCR mice at a ratio of 1:1. The data shown are the frequencies of divided and undivided cells, as represented by the expression of CFSE determined using flow cytometry. The data shown represent the means ± SD of four to seven biological replicates per experiment (**A–E**), technical replicates combined over two experiments (**F**), or technical replicates from a representative experiment (**G**). Significant differences are indicated with asterisks (*P < 0.05; **P < 0.01; ***P < 0.001) by Kruskal-Wallis test with Dunn’s multiple comparisons tests (**A–G**).

After *Mtb* infection, T cell priming is initiated in the draining lymph nodes (DLNs), where *Mtb* antigens are presented by migrating mDCs (14, 15). To assess whether the impairment of T cell activation was linked to defects in DC-mediated *Mtb* transport to the DLNs, we enumerated *Mtb* burden in the DLNs early in infection at 14 dpi. We found significantly lower *Mtb* colony-forming unit (CFU) in the DLNs of mice infected with *rpoB*-H445Y *Mtb* compared to *Wt* HN878 *Mtb* (Fig. 4A). At this early time point, we also found decreased accumulation of MHC Class II-expressing mDCs in the lungs of *rpoB*-H445Y *Mtb* infection (Fig. 4B). These results suggest that the reduction in accumulation of activated T cells in the lung might be due to reduced activation of mDCS and trafficking of *Mtb*-infected lung mDCs to the DLNs. B6 mice were infected with *Wt* or *rpoB*-H445Y *Mtb* expressing mCherry reporter, and while there were no differences in the number of *Mtb* on a per cell basis in mDCs, there was decreased frequency of *rpoB*-H445Y *Mtb-infected* mDCs in contrast to *Wt Mtb*, which was mainly harbored within mDCs (12, 15) (Fig. 4D and E). To address if the *rpoB-H445Y Mtb* infection differentially impacted the migratory potential of mDCs toward chemokines (e.g., CCL-19), we used an *in vitro* chemotaxis assays and found that *rpoB-*H445Y *Mtb-*infected bone marrow-derived dendritic cells (BMDCs) did not migrate as effectively as *Wt Mtb*-infected BMDCs (Fig. 4F). In a co-infection model where BMDCs were infected with a 1:1 ratio of *wt* and *rpoB*-H445Y *Mtb*, the BMDCs recapitulated the limited migration observed in *rpoB-*H445Y *Mtb* infection. These results suggest that the immune suppression driven by *rpoB-*H445Y *Mtb* dominates and limits DC migration. We also sought to assess whether *rpoB-*H445Y *Mtb* infection of DCs impairs T cell activation *in vitro* by co-culturing naïve CD4^+^ T cells from *Rag* ^−/−^ ESAT6_1-20_ TCR transgenic mice that recognize *Mtb* antigen with BMDCs were infected with either *Wt Mtb* or *rpoB*-H445Y *Mtb*. Interestingly, T cell proliferation was observed to similar levels independent of the *Mtb* strain (Fig. 4G). Together, our results suggest that the process of infected mDCs translocating *Mtb* to the DLNs for priming of T cells is impaired in mice infected with *rpoB-*H445Y *Mtb* infection.

### Type I IFN signaling suppresses myeloid and lymphoid cell recruitment and activation during rpoB-H455Y *Mtb* infection

**Fig 5:**
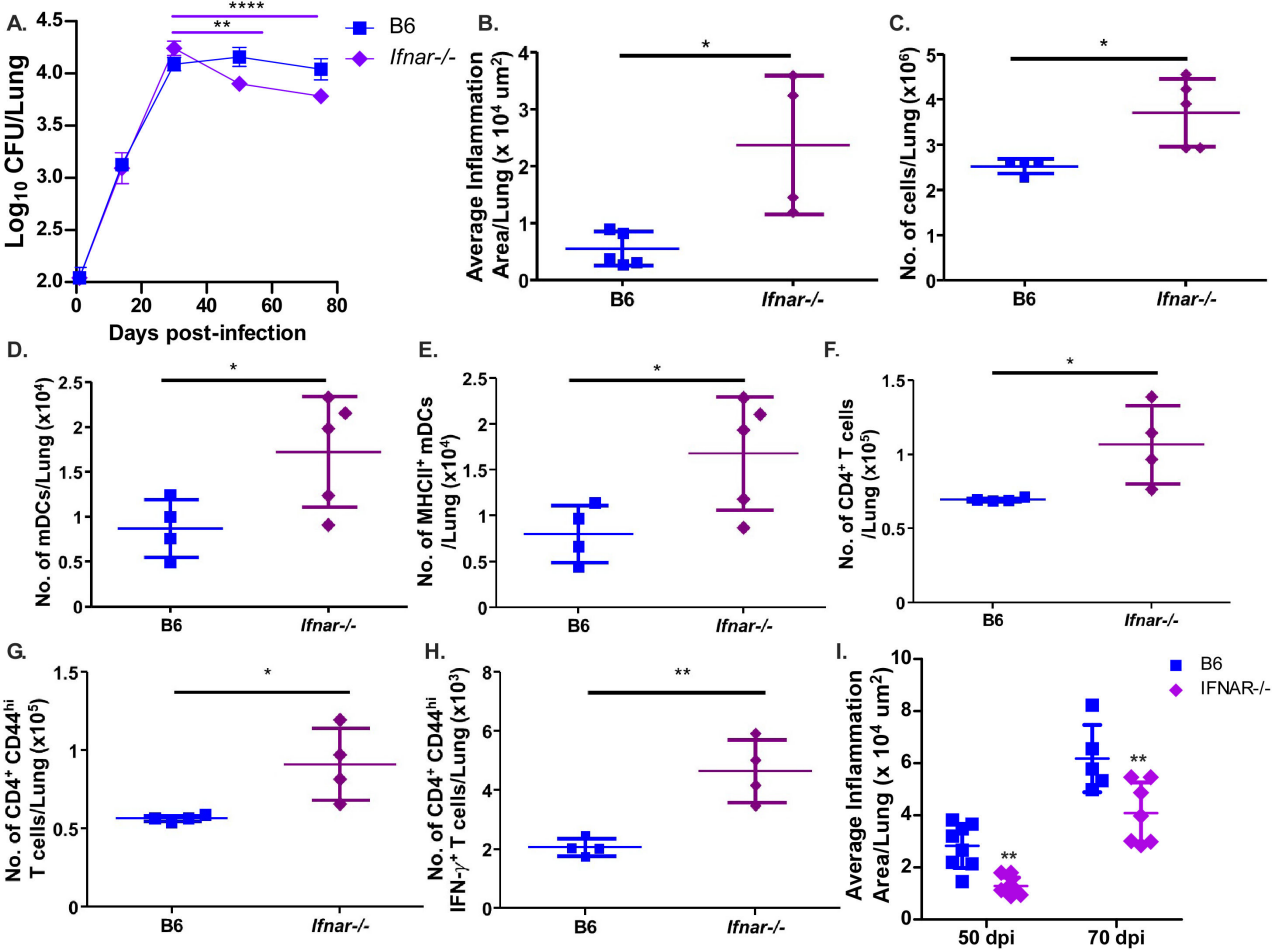
*rpoB*-H445Y *Mtb* infection suppresses lung immune responses through a type I IFN-dependent pathway. B6 and *Ifnar* ^−/−^ mice were aerosol infected with a low dose of *rpoB*-H445Y *Mtb*. Mice were sacrificed for subsequent analyses at the indicated time points. Bacterial burden in (**A**) lung was determined. (**B**) FFPE lung sections were stained with H&E, and lung inflammatory area was measured at 30 dpi. (**C**) Total numbers of lung immune cells in single cell suspensions were determined at 30 dpi. (**D**) Total numbers of (**E**) MHC Class II^+^ mDCs, as well as the numbers of (**F**) total, (**G**) CD44^+^, and IFN^+^ (**H**) CD4^+^ T cells, were determined by flow cytometry at 30 dpi. (**I**) FFPE lung sections were stained with H&E, and lung inflammatory area was measured at 50 and 70 dpi. The data shown represent the means ± SD of four to five biological replicates per experiment. Significant differences are indicated with asterisks (*P < 0.05; **P < 0.01; *P* < 0.0001) by Kruskal-Wallis test with Dunn’s multiple comparisons tests (**A–H**) or by two-way ANOVA with Bonferroni post test (**I**). One of two independent experiments is shown.

From our cytokine data, we hypothesized that type I IFN production was playing a role in the induction of immune responses following infection. Thus, B6 mice and mice lacking the IFN-α/β receptor (*Ifnar^−/^* ^−^) were infected with *rpoB*-H445Y *Mtb*. While there were no differences in *Mtb* burden in the lungs between B6 and *Ifnar^−/−^ rpoB*-H445Y *Mtb-*infected mice at 30 dpi (Fig. 5A), lung inflammation and immune cell accumulation were increased in *rpoB*-H445Y *Mtb*-infected *Ifnar^−/^* ^−^ mice (Fig. 5B and C). Importantly, accumulation of mDCs and other myeloid subsets, including RMs and monocytes, was also increased in *Ifnar^−/−^ rpoB*-H455Y *Mtb*-infected mice (Fig. 5D; Fig. S5A and B). Additionally, the number of activated mDCs that expressed MHC Class II was also increased in *Ifnar^−/−^ rpoB*-H455Y *Mtb*-infected lungs when compared to their B6 counterparts (Fig. 5E). In contrast, when we infected B6 and *Ifnar^−/^* ^−^ mice with *wt* HN878 *Mtb*, we found equivalent lung and splenic bacterial burden and no differences in inflammation and total immune cell accumulation between the lungs of these two groups of mice (Fig. S6A through D). The numbers of total and MHC Class II-expressing RMs and monocytes were not significantly different between B6 and *Ifnar^−/^* ^−^ mice infected with *wt* HN878 *Mtb* (Fig. S5A through D). Next, we addressed whether improved mDC activation in *Ifnar^−/^* ^−^ mice infected with *rpoB*-H455Y *Mtb* also improved activation of T cell responses. Indeed, we found that *Ifnar^−/−^ rpoB*-H445Y *Mtb*-infected mice accumulated significantly higher numbers of total CD4^+^ T cells, as well as activated CD4^+^CD44^hi^ T cells, when compared with B6 mice infected with *rpoB*-H445Y *Mtb* (Fig. 5F and G). The absence of type I IFN signaling also increased *Mtb*-specific IFN-producing CD4^+^ T cells in *rpoB*-H445Y *Mtb* infection (Fig. 5H). Together, these data demonstrate that H445Y and other associated SNPs in *Mtb* and its impact on bacterial determinants may suppress inflammation, immune cell recruitment, as well as myeloid cell activation and T cell responses through a type I IFN-dependent mechanism during *Mtb* infection. This suppression of the immune response modulated through type I IFN signaling appears to benefit *rpoB*-H445Y *Mtb* as *Ifnar^−/^* ^−^ mice show a decrease in lung bacterial burden during the chronic stages of infection with this DR mutant (Fig. 5A). Coinciding with this decrease in bacterial loads, *rpoB*-H445Y *Mtb*-infected *Ifnar^−/^* ^−^ mice also showed a decrease in the average size of inflammatory lesions as compared with their B6 counterparts (Fig. 5I). Thus, our results propose that the H445Y SNP may result in heightened type I IFN production in mice, which dampens the initial immune response and leads to increased bacterial abundance and worse pathology for the host.

## DISCUSSION

The rise of drug resistance has exacerbated efforts to combat and contain TB. Clinical reports have suggested that, in addition to being difficult to treat, MDR TB patients also exhibit dysregulated immune responses (2 – 5). Despite extensive studies that have described the effects of DR mutations in *rpoB* on rifampicin resistance and bacterial fitness (16), the impact of these SNPs on the host response has been unexplored and poorly understood. In our previous study, we showed that the presence of specific rifampicin drug-resistant mutations alters macrophage responses to *Mtb* infection *in vitro* through changes in bacterial cell wall lipid abundance and structure (10). In this work, we studied the association between drug-resistance-conferring mutations and *in vivo* host responses with the two most common mutations that account for nearly 90% of rifampicin-resistant MDR TB infections annually (6 – 9). While both *Mtb* strains, *rpoB-*H445Y and *rpoB-*S450L, are resistant to rifampicin, they are associated with vastly differing host responses during infection in the lung. Importantly, we found broader impacts on immunopathology of TB disease that are not attributable to bacterial burden. More specifically, we observed limits in mDC trafficking, which resulted in impairment of T cell generation and limited inflammation. This immunomodulation in the lung was achieved through a host type I IFN-dependent pathway, and removing this signaling not only lifted the suppression of the immune response but also led to improved control of *Mtb* during chronic infection. Together, our results demonstrate that infection with *Mtb* strains carrying drug-resistance-conferring and associated mutations results in divergent host immune responses and highlights that these responses have a regulatory role in immunopathogenesis *in vivo*.

Type I IFN signaling has a controversial role in *Mtb* infections. Individuals with TB disease and TB progressors show upregulation of a type I IFN transcriptional signature (17 – 20). In mice, type I IFN signaling has been shown to play a counter-regulatory role with IL-1 signaling and suggested to drive DS *Mtb* susceptibility by antagonizing IL-1 production and signaling (21 – 24). Conversely, other studies suggest that IFN-β may have a protective role through the induction of nitric oxide in infected macrophages (25, 26). Our study sheds light on a novel role for type I IFN signaling, specifically in some DR *Mtb* infections. We observed that *rpoB-*H445Y *Mtb* infection results in a heightened type I IFN production that suppressed the recruitment of activated immune cells to the site of infection and the downstream initiation of adaptive immune responses. This immune modulation appears to be important for *Mtb* pathogenesis, as lack of type I IFN signaling reversed innate and adaptive immune responses, leading to an enhanced bacterial control during chronic *rpoB*-H445Y *Mtb* infection. Therefore, induction of type I IFNs and suppression of host immune responses could serve as a mechanism for some DR *Mtb* strains to persist in the host. This finding stands in stark contrast to DS *Mtb* infection, where lack of type I IFN signaling had no discernable impact on inflammation or cell recruitment at the same time points.

In previous studies, the role of type I IFN signaling in DS *Mtb* infections has been discernible in hypersusceptible mouse strains (27, 28); hyper-IFN induction using poly IC or recombinant type I IFN co-treatment with *Mtb* infection (29, 30); or blockade of GM-CSF, a key mediator of *Mtb* immunity (31). In these studies, the observed role for type I IFNs was to drive the recruitment of *Mtb-*permissive cells and promote pathology, thus promoting TB disease. While type I IFN signaling during *rpoB*-H445Y *Mtb* infection also promoted *Mtb* pathogenesis, type I IFN signaling plays the opposite functions in that it suppressed immune responses and limited pathology. Also, our data suggest that heightened type I IFN signaling during *rpoB*-H445Y *Mtb* infection is not influencing the permissibility or frequency of apoptosis of the cells in which the DR *Mtb* is harbored. Additionally, type I IFN signaling did not impact T cell responses in DS *Mtb* infection of hypersusceptible mice (27), while during *rpoB*-H445Y *Mtb* infection, the restricted mDC accumulation results in reduced T cell responses through IFNAR-dependent pathways. It has been suggested that the hypervirulence of the DS *Mtb* clinical isolate, HN878, used in this study is tied to a limited induction of Th1 and associated with greater type I IFN induction, relative to other DS *Mtb* strains (30, 32). Here, we showed a link between heightened type I IFN induction and restricted Th1 immunity during infection with our *rpoB*-H445Y *Mtb* strain. Together, our findings suggest that, although type I IFN signaling might have a detrimental role in both *Mtb* infections, the detrimental effects observed in *rpoB*-H445Y DR *Mtb* infection are likely through distinct pathways.

There are a few potential explanations for the discrepancies between our findings and previously published work regarding the mechanism of action of type I IFN signaling between the DS and DR *Mtb* infections in the study. The nature of the H445Y mutation in *rpoB* does alter the transcriptional landscape and the production of various virulence factors in *Mtb* (16). We have previously shown that the presence of this specific H445Y SNP in *rpoB* is associated with changes to the bacterial cell wall lipid composition and abundance, which are sufficient to drive differential cytokine production in macrophages (10). Similarly, other important virulence factors that affect the impact of type I IFN signaling in cells infected with DS *Mtb* could be differentially transcribed in *rpoB-*H445Y DR *Mtb*. Banks and colleagues noted that DS *Mtb* inhibits type I IFN signaling in infected macrophages in a dose-dependent manner to promote intracellular survival (25). The addition of supraphysiological levels of IFN overcame *Mtb* suppression of type I IFN signaling and decreased bacterial loads. Various IFN-induced genes noted to be transcriptionally inhibited by DS *Mtb*, such as *Ifit1, Ccl12*, and *Iigp1*, were found to be transcriptionally upregulated in MDR *Mtb*-infected macrophages, relative to those infected with DS *Mtb* (10, 25). These findings suggest that DR and MDR *Mtb* with the H445Y SNP might not be inhibiting autocrine type I IFN signaling, potentially through decreased transcription of phosphatases known to interfere with various host signaling pathways (33). Thus, in the absence of these *Mtb* virulence factors, heightened type I IFN signaling could drive distinct outcomes between DS *wt* and *rpoB*-H445Y *Mtb* infections.

Other explanations can be found on the host side of this host-pathogen interaction. Desvignes and colleagues found a protective role for type I IFN signaling in the absence of type II IFN signaling by limiting the influx of permissive myeloid cells (34). Mice lacking both, type I and type II IFN, signaling pathways had exacerbated lung histopathology and succumbed to infection earlier. In the context of our study, we found fewer type II IFN-producing T cells during *rpoB*-H445Y *Mtb* infection, suggesting a lung environment with limited IFN. Therefore, the differences in the production and subsequent signaling of type II IFNs between DS and *rpoB-*H445Y *Mtb* could set the stage for different roles of type I IFN in *Mtb* infections. Another explanation for the different roles of type I IFN between the two *Mtb* infections is that the two strains of *Mtb* are interacting with different cell types. Our findings suggest that, while DS *wt Mtb* is mainly found in mDCs, *rpoB*-H445Y *Mtb* is not harbored to the same degree in this cell type. If *rpoB*-H445Y *Mtb* is found to be in different host cells than DS *wt Mtb*, then induction of type I IFN signaling in these other cell types could drive different cellular responses and not result in increased infiltration of permissive cells and loss of control of the infection.

Our study opens up several questions which need to be addressed to fully understand the pathogenesis of RDR and MDR TB. How does type I IFN signaling limit myeloid cell activation and recruitment as seen by us and others (34)? We observed a decrease in the levels of a few chemokines and cytokines in *rpoB*-H445Y *Mtb* infection of B6 mice and human MDMs, respectively, but it remains to be understood the pathways linking type I IFN signaling and inhibition of chemokine production and MHC Class II. By limiting the production and activity of potent pro-inflammatory molecules, type I IFNs could be curtailing the inflammatory cell recruitment in *rpoB*-H445Y *Mtb* infection. Similarly, how does *rpoB*-H445Y *Mtb* infection impair DC migratory ability? From the reduced accumulation of *rpoB*-H445Y *Mtb* in the DLNs early in infection and the decreased chemotactic sensitivity of infected BMDCs to CCL19, DCs infected with *rpoB*-H445Y *Mtb* likely have diminished expression of the cognate receptor, CCR7. Furthermore, a co-infection with an equal ratio of DS *wt* and *rpoB*-H445Y *Mtb* reveals that the suppressed chemotaxis response of *rpoB*-H445Y *Mtb* is dominant. Together, these data suggest that the *rpoB*-H445Y *Mtb* strain might suppress DC activity through a secreted virulence factor or heightened type I IFN signaling.

Although our study reveals intriguing insights into the intersection between drug resistance and host immunity, there are a few limitations of the work. One such limitation is the lack of matched mutant strains and the use of a complemented strain. While we generated the mutant RDR *Mtb* strains with the use of the *wt Mtb* strain, a few additional SNPs exist that separate the *wt Mtb* strain from the RDR *Mtb* strains beyond the RDR-conferring SNPs in *rpoB*. Despite our best efforts to genetically engineer the specific point mutation in *rpoB* in the *wt Mtb* strain or to generate a complemented *Mtb* strain with the *wt* sequence in the *rpoB*-H445Y *Mtb* strain, these tasks have proved to be technically difficult to execute. As such, it is possible that other SNPs outside of H445Y in *rpoB* could contribute to the phenotypes observed in the study. However, in our collection of independently generated *rpoB* mutant strains, we observed the repeated occurrence of these additional SNPs throughout the panel. This suggests that these mutations may be commonly co-occurring spontaneous mutations generated alongside *rpoB* mutations. Moreover, the presence of these additional SNPs did not phenotypically impact *Mtb* infection of BMDMs with strains not containing the *rpoB*-H445Y SNP. Yet, the question of causality with regard to the specific *rpoB* mutations and the observed phenotypes still remains open and needs to be confirmed in future studies. We also know little about how SNPs in *rpoB* lead to altered host-pathogen interactions. Previous studies have suggested that compensatory transcriptional changes might occur in *Mtb* strains as a result of the *rpoB* SNPs (27). Additionally, the location of these SNPs in the *rpoB* could also drive differences in transcription. However, the nature of these transcriptional changes and how they are linked to the phenotypes observed in the study remain to be determined.

In summary, our studies have mechanistically shown how the identities of the drug-resistance-conferring mutations are associated with differential host responses to infection and the relationship between drug resistance and TB disease pathogenesis. These altered host responses can be leveraged by the drug-resistant bacteria to promote its survival. The implications of these diverse immune responses underscore the importance of appreciating bacterial heterogeneity in studying host-pathogen interactions. While much of the focus of the field in studying host immunity to TB has centered around host factors and background, our study emphasizes the need to also approach the issue from the bacterial perspective. Fully delineating such immune mechanisms of antagonism by RDR *Mtb* infection and specific DR mutations in the future will allow us to develop targeted host-directed therapeutics and vaccines addressing the growing incidence of RDR and MDR TB.

## MATERIALS AND METHODS

### Mice

C57BL/6J (B6), C3HeB/FeJ, and *Ifnar* ^–/–^mice on the B6 background were purchased from The Jackson Laboratory (Bar Harbor, ME, USA). ESAT-6 TCR transgenic mouse line was established as previously described and maintained on the *Rag1^−/−^* background, with the αβ T cells recognizing IA^b^/ESAT6_1-20_ (11, 35). All mice were maintained in the animal facility at Washington University in St. Louis and bred in-house. Experimental mice were age and sex matched and infected between the ages of 6 and 8 weeks. All mice were maintained and used in accordance with the approved Washington University in St. Louis Institutional Animal Care and Use Committee guidelines.

### Generation of BMDMs and BMDCs

Bone marrow-derived macrophages and bone marrow-derived dendritic cells were generated as previously described (10, 12). Briefly, bone marrow cells were collected from the tibia and femur of B6 mice and cultured in complete Dulbecco’s modified Eagle’s medium (cDMEM) with 20 ng/mL of recombinant granulocyte-macrophage colony-stimulating factor (GM-CSF). Cells were cultured at 37°C in 5% CO_2_ and supplemented with media on day 3. On day 7, non-adherent cells were collected as BMDCs and adherent cells were collected as BMDMs.

### Generation of human monocyte-derived macrophages

Peripheral blood was obtained from the Mississippi Valley Regional Blood Center in LRS chambers through the Fehniger Laboratory. LRS chambers were flushed with PBS containing heparin (1 U/mL) and 2% HuAB (Sigma H3667-100ML, Lot #SLBZ1915) to collect blood. The diluted blood was then overlaid onto Ficoll (Lymphoprep, AXS-1114544, Axis Shield) and centrifuged to isolate peripheral blood mononuclear cells. CD14^+^ cells were enriched using CD14 Microbeads (130-050-201, Miltenyi Biotech) according to the manufacturer’s instructions. Cells were cultured in cRPMI1640 containing 750 IU/mL of human GM-CSF (Miltenyi Lot #5180329061) at 37°C in 5% CO_2_, with media changes every 2–3 days as needed for 7 days.

### *Mtb* infections

*Mtb* strain HN878 was obtained from BEI resources (Manassas, VA, USA) under National Institutes of Health contract AI-75320. HN878-mCherry was generated by transforming the *Mtb* strain HN878 with a plasmid (pMSG432) encoding mCherry. Independent rifampicin-resistant *Mtb* HN878 colonies (biological replicates) were selected from rifampicin (2 µg/mL) containing 7H11 agar plates (36). The sequences of *rpoB* were confirmed by Sanger sequencing (Genewiz), and *Mtb* stocks were created for further experimentation. All *Mtb* strains were cultured in Proskauer Beck medium supplemented with 0.05% Tween 80 and frozen at −80°C while in mid-log phase. Colony forming units of the bacterial stocks were determined through serial dilutions on 7H11 agar plates. Mice were aerosol infected with low doses (~100 CFU) of indicated *Mtb* strains in sterile PBS using a Glass-col nebulizer (37). *Mtb* bacterial burden/organ was quantitated by plating serial dilutions of homogenized lung, spleen, or lymph node tissue or aspirated bone marrow on 7H11 agar plates (BD Biosciences, Franklin Lakes, NJ, USA). Plates were incubated for 2–3 weeks at 37°C, and the number of colonies was counted. *In vitro* infections were carried out at a multiplicity of infection (MOI) of 1 in respective antibiotic-free media for the indicated number of days.

### Flow cytometry

Lungs were perfused with heparin in PBS, minced, digested with DNAse/collagenase, lysed for red blood cells, and pressed through a 70-µm filter to generate a single-cell suspension (37). For quantification of cytokine responses, single-cell suspensions were stimulated *ex vivo* with either Phorbol Myristic Acid, Ionomycin, and GolgiStop (BD Biosciences) for 5 h or purified *Mtb* antigens ESAT6 and Ag85B (10 µg/mL) overnight along with brefeldin and monensin (BD Biosciences) as previously described (38, 39). Cells were treated with Fc Block (CD16/CD32,2.4G2, Tonbo Biosciences) and stained with appropriate fluorochrome-labeled specific antibodies or isotype control antibodies. Intracellular cytokine staining was performed using the BD Cytofix/Cytoperm kit (BD Biosciences). Mouse antibodies used include CD11b (M1/70; Tonbo Biosciences), CD11c (HL3; BD Biosciences), Gr-1 (RB6–8C5, eBioscience), CD3 (500A2; BioLegend), CD4 (RM4–5; BD Biosciences), CD44 (IM7; Tonbo Biosciences), IFN (XMG1.2; Tonbo Biosciences) or isotype control IgG1 (A85-1; BD Biosciences), CD8 (53-6.7; BD Biosciences), MHC Class II (M5/114.15.2; Tonbo Biosciences), Ki-67 (16A8; BioLegend) or isotype control IgG2a, (RTK2758; BioLegend), 7AAD (BD Biosciences), and Annexin V (BD Biosciences).

Cells were processed with the Becton Dickinson (BD) Fortessa X-20 flow cytometer using FACS Diva software or the BD FACSJazz flow cytometer using FACS software (BD). Flow cytometry experiments were analyzed using FlowJo (Tree Star Inc.). As before (10, 12), monocytes were defined as CD11b^+^CD11c^−^Gr-1^med^ cells, and recruited macrophages were defined as CD11b^+^CD11c^−^Gr-1^low^ cells. mDCs were defined as CD11b^+^CD11c^+^ cells. T cells were defined as CD3^+^CD4^+^ or CD3^+^CD8^+^ cells. Total numbers of cells within each gate were back calculated based on cell counts/individual sample.

### Histology

Lung lobes were perfused with 10% neutral buffered formalin and embedded in paraffin. Formalin-fixed paraffin-embedded (FFPE) lung sections were stained with hematoxylin and eosin (H&E), and inflammatory features were evaluated by light microscopy. Inflammatory lesions were outlined with the automated tool of the Zeiss Axioplan 2 microscope (Carl Zeiss), and the total inflammatory area in each lung lobe was measured.

### Determination of proteins

Cytokine and chemokine production in the lung homogenates of *Mtb-*infected mice and the supernatants of *Mtb-*infected MDMs was analyzed using Milliplex Multiplex Assays (Millipore) according to the manufacturer’s protocol. IL-1 and IFN were measured using Quantikine ELISA kits (R&D Systems), following the manufacturer’s instructions.

### Chemotaxis assay

BMDCs, generated using the above protocol, were infected, with their respective strain, at an MOI of 1 in antibiotic-free cDMEM. For co-infections, the amount of *Mtb* was titrated to the ratios specified (*Wt Mtb:rpoB-*H445Y *Mtb*). Chemotaxis was carried out as described previously (12, 40). Briefly, 1 dpi, BMDC migration in response to CCL-19 was determined through flow cytometry, using a standard number of fluorescent beads (Polysciences Inc.) to normalize the samples. The results are indicated as chemotaxis index, which is represented as the fold increase in the number of migrated cells in each sample, relative to spontaneous migration in the gravity control group.

### T cell proliferation assay

BMDCs were similarly generated and infected for 1 day with the respective *Mtb* strain at an MOI of 1. T cell co-culture proliferation assay was carried out as previously described (11). Briefly, transgenic CD4^+^ T cells recognizing IA^b^/ESAT-6_1–20_ were isolated from the spleens and lymph nodes of mice, enriched using CD4^+^ T cell MACS Microbeads (Miltenyi Biotech) and then stained with carboxyfluorescein succinimidyl ester (CFSE) for 15 min at 37°C. Infected or uninfected BMDCs and naïve, CFSE-labeled T cells were co-cultured with recombinant IL-2 (ThermoFisher) for 6 days. Cells were then stained and processed as described above.

### Bacterial genomic DNA isolation and sequencing

In brief, *Mtb* cultures were expanded to mid-log phase and lysed through bead beating, and genomic DNA was extracted using phenol-chloroform-isoamyl alcohol. DNA was precipitated with isopropanol at −20°C overnight. DNA pellet was washed in 80% ethanol and dissolved in nuclease-free water. Genomic libraries were prepared using WGS KAPA Hyper-PCR free method and sequenced using the NovaSeq platform to target 15 Gb (GTAC MGI).

### Whole-genome sequence data processing, variant calling, and chromosome visualization

Raw paired-end fastq reads were filtered and trimmed with Trim Galore v0.4.1 (Babraham Bioinformatics) and mapped to the H37Rv reference genome (NC_000962.3) using Burrows-Wheeler Aligner v0.7.12 (41). Samtools v1.5 (42) were used for removing duplicates and indexing with default settings. SNPs were then identified and filtered using Samtools v1.5 mpileup (42) and Bcftools v1.5 (42). SNPs with low quality (QUAL ≤100), low read consensus (≤75% reads supporting alternate allele), and high proximity to indels (within 15 bp) were filtered out. Additionally, polymorphisms in or within 50 base pairs of hypervariable PPE/PE gene families, repeat regions, and mobile elements were excluded. The resulting VCF files were used for SNP density visualization in R studio with package BioCircos (43) and Gviz (44).

### Statistical analysis

All data sets were evaluated for normality using the Shapiro-Wilk test. Differences between the means of groups were analyzed using either the two-tailed Student’s *t*-test or the Mann Whitney *U* test, where applicable. Differences between the means of more than two groups were analyzed using one-way ANOVA or two-way ANOVA for time course studies, with Tukey’s post-tests for normally distributed distributions or Kruskal-Wallis test with Dunn’s multiple comparisons tests. All statistical analyses were done in GraphPad Prism 9. A *P* value <0.05 was considered significant. The data points across figures represent the mean (±SD or +SEM) of values as noted. ^*^ *P* ≤ 0.05; ^**^ *P* ≤ 0.01; ^***^ *P ≤* 0.001; ^****^ *P ≤* 0.0001; ns, not significant (*P* > 0.05). All experiments were replicated for reproducibility.

## Data Availability

All data that support the findings of this study are available from the corresponding author upon request. DNA sequencing data have been submitted under BioProject ID PRJNA811702.

## SUPPLEMENTAL MATERIAL

Fig. S1 mbio.00946-23-s0001.png Mtb isolates containing rpoB-H445Y SNP drive altered type I interferon production during infection.

Fig. S2 mbio.00946-23-s0002.tif
*rpoB*-H445Y *Mtb* infection limits myeloid cell recruitment and activation in mice.

Fig. S3 mbio.00946-23-s0003.tif
*rpoB*-H445Y *Mtb* infection induces altered cytokine and chemokine production in mice.

Fig. S4 mbio.00946-23-s0004.tif
*rpoB*-H445Y *Mtb* infection also results in a limited immune response in FeJ mice.

Fig. S5 mbio.00946-23-s0005.tif
*Ifnar* ^-/-^ mice have significantly enhanced myeloid cell recruitment and activation in the lung only after infection with *rpoB*-H445Y *Mtb*.

Fig. S6 mbio.00946-23-s0006.png Absence of type I IFN signaling does not impact wt HN878 *Mtb* infection.

Supplemental Figure Legends mbio.00946-23-s0007.docx Legends for all supplemental figures.

ASM does not own the copyrights to Supplemental Material that may be linked to, or accessed through, an article. The authors have granted ASM a non-exclusive, world-wide license to publish the Supplemental Material files. Please contact the corresponding author directly for reuse.
