# Supplementary figures and images for: Mycobacterium tuberculosis carrying the rifampicin drug-resistance-conferring rpoB mutation H445Y is associated with suppressed immunity through type I interferons
Source: mBio. 2023 Sep 8;14(5):e00946-23. doi: 10.1128/mbio.00946-23 (PMC10653897; doi:10.1128/mbio.00946-23)

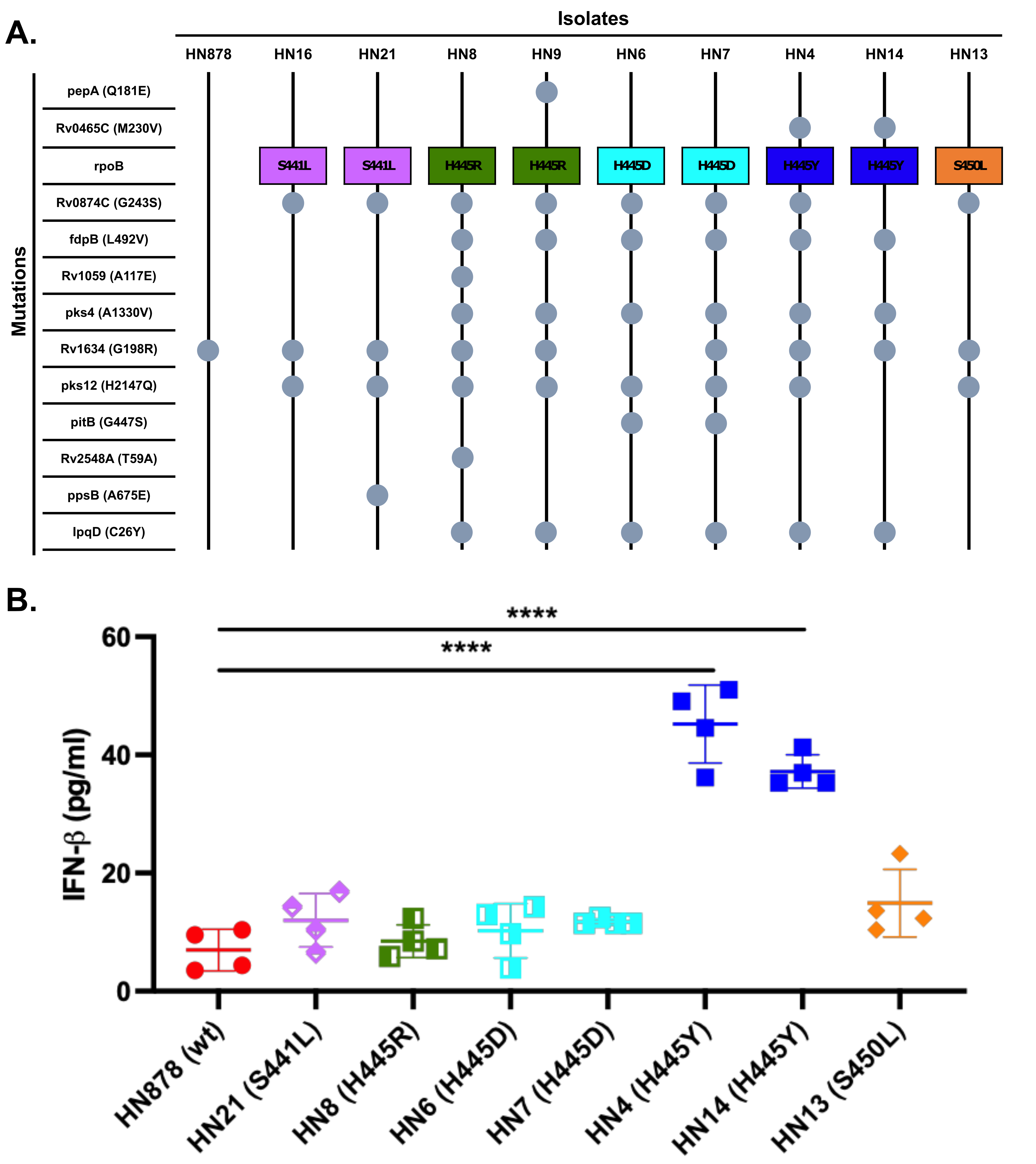

Supplement: Fig. S1 — Mtb isolates containing rpoB-H445Y SNP drive altered type I interferon production during infection. [file mbio.00946-23-s0001.png]

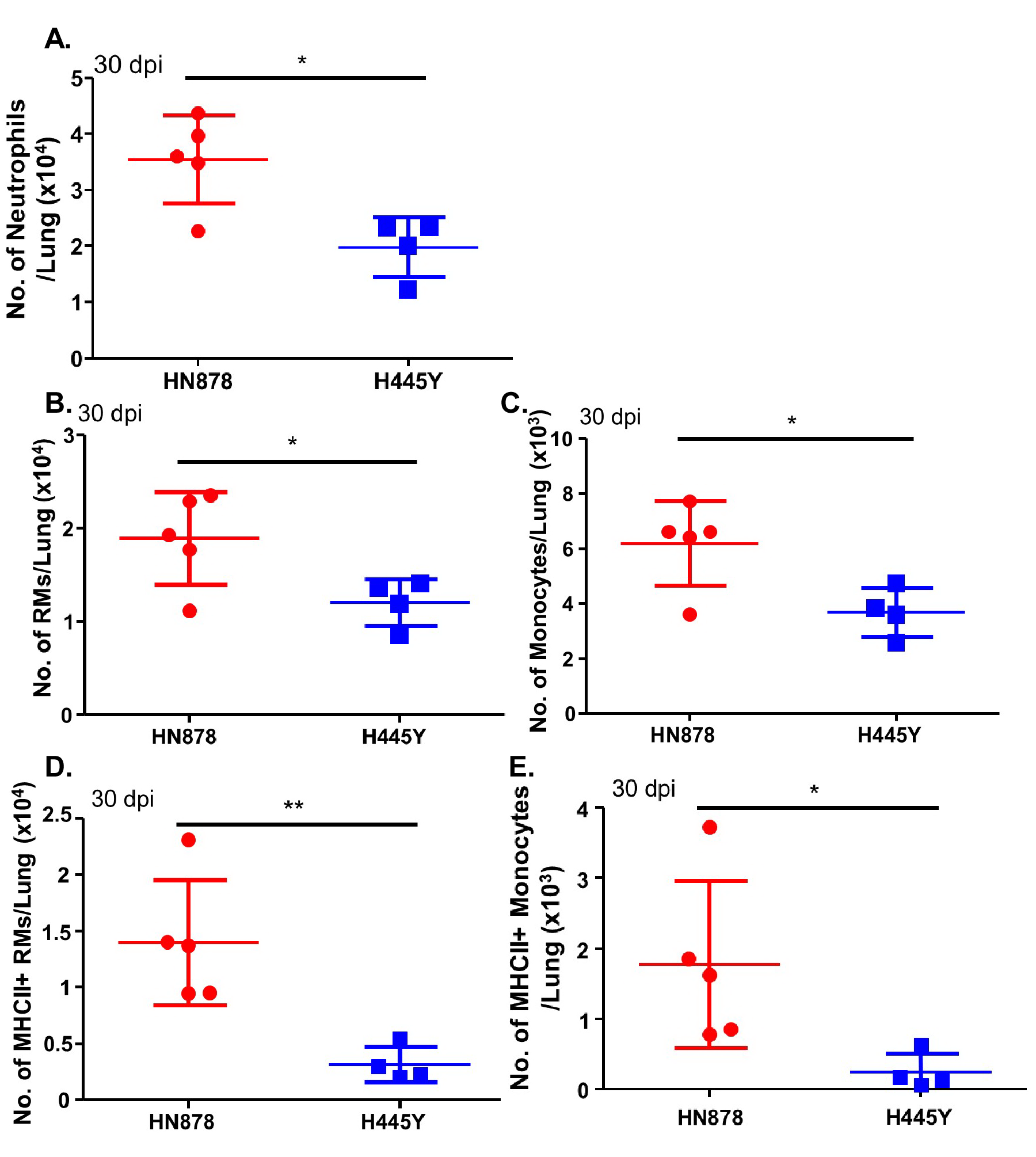

Supplement: Fig. S2 — rpoB-H445Y Mtb infection limits myeloid cell recruitment and activation in mice. [file mbio.00946-23-s0002.tif]

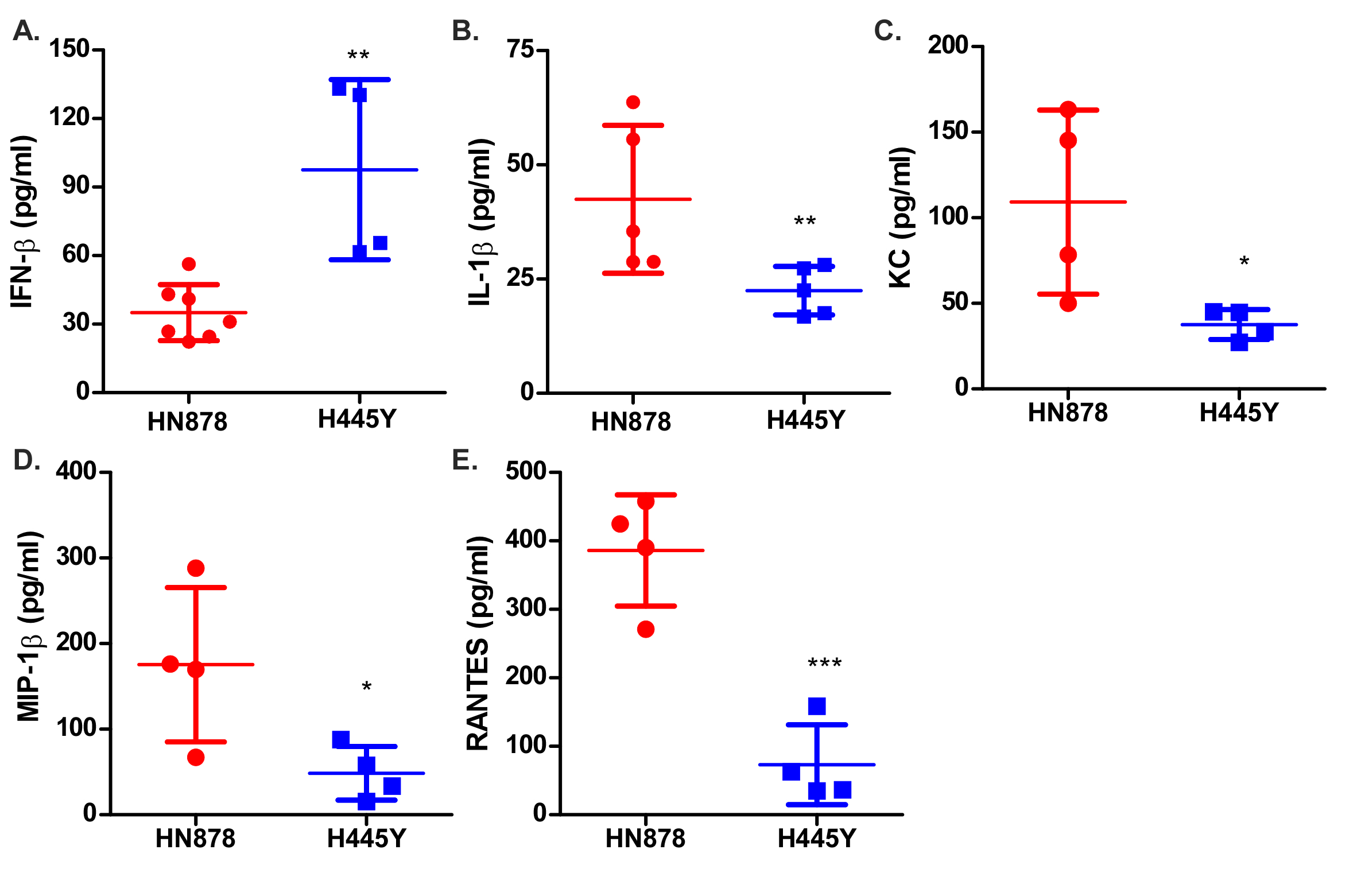

Supplement: Fig. S3 — rpoB-H445Y Mtb infection induces altered cytokine and chemokine production in mice. [file mbio.00946-23-s0003.tif]

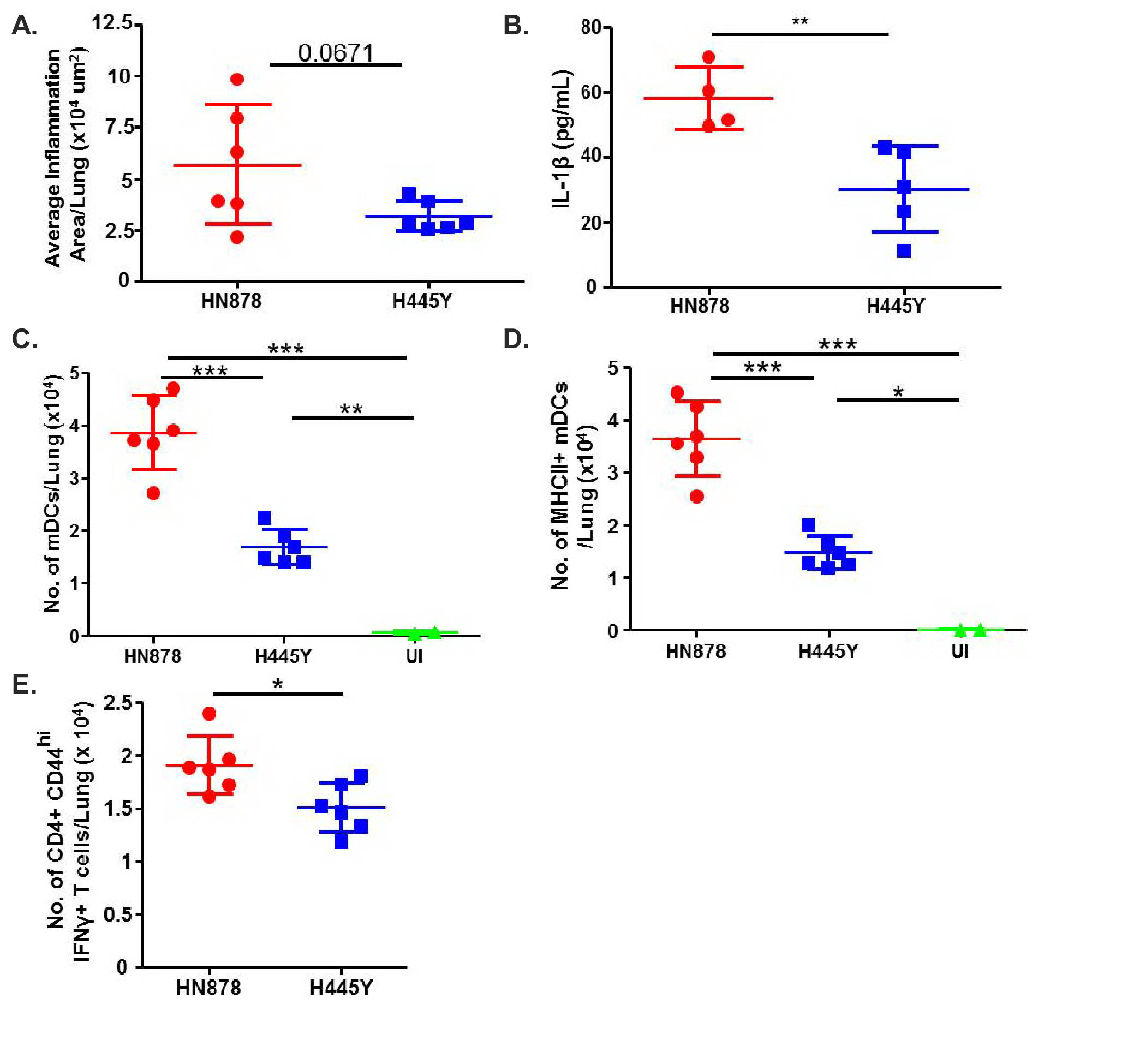

Supplement: Fig. S4 — rpoB-H445Y Mtb infection also results in a limited immune response in FeJ mice. [file mbio.00946-23-s0004.tif]

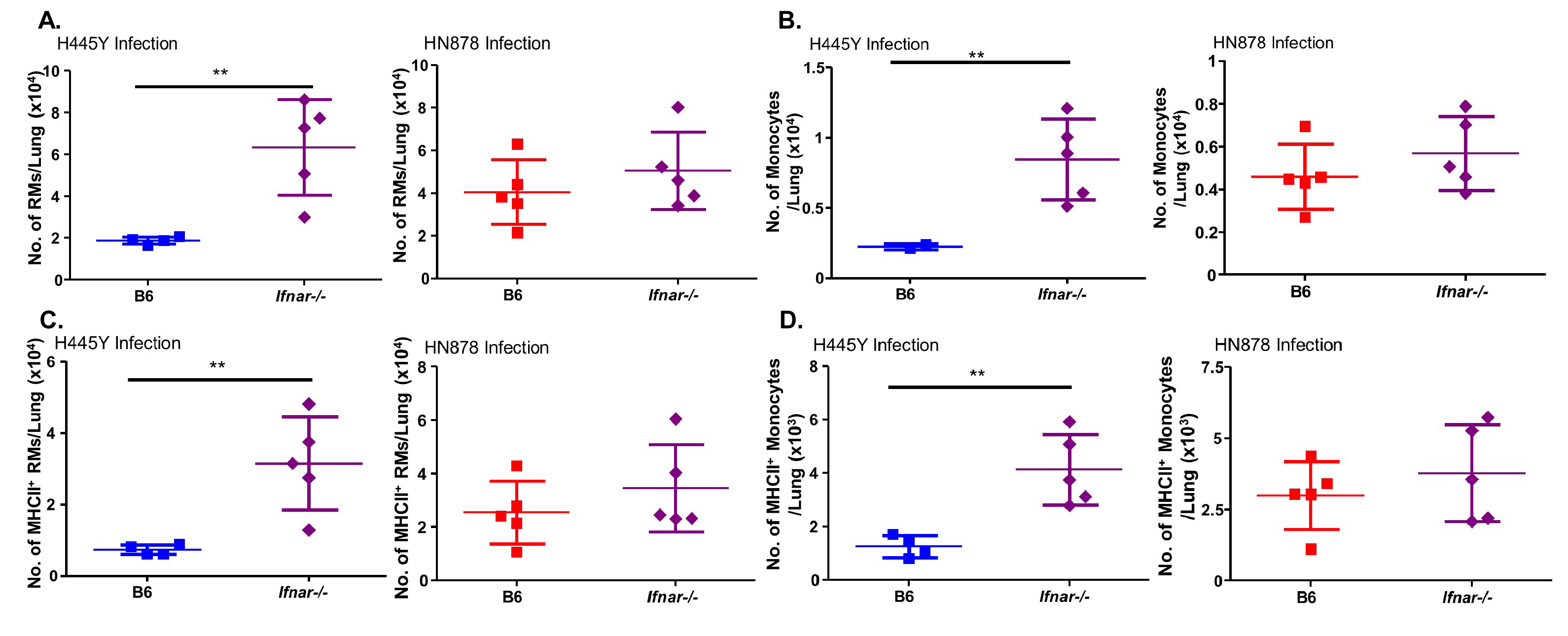

Supplement: Fig. S5 — Ifnar -/- mice have significantly enhanced myeloid cell recruitment and activation in the lung only after infection with rpoB-H445Y Mtb. [file mbio.00946-23-s0005.tif]

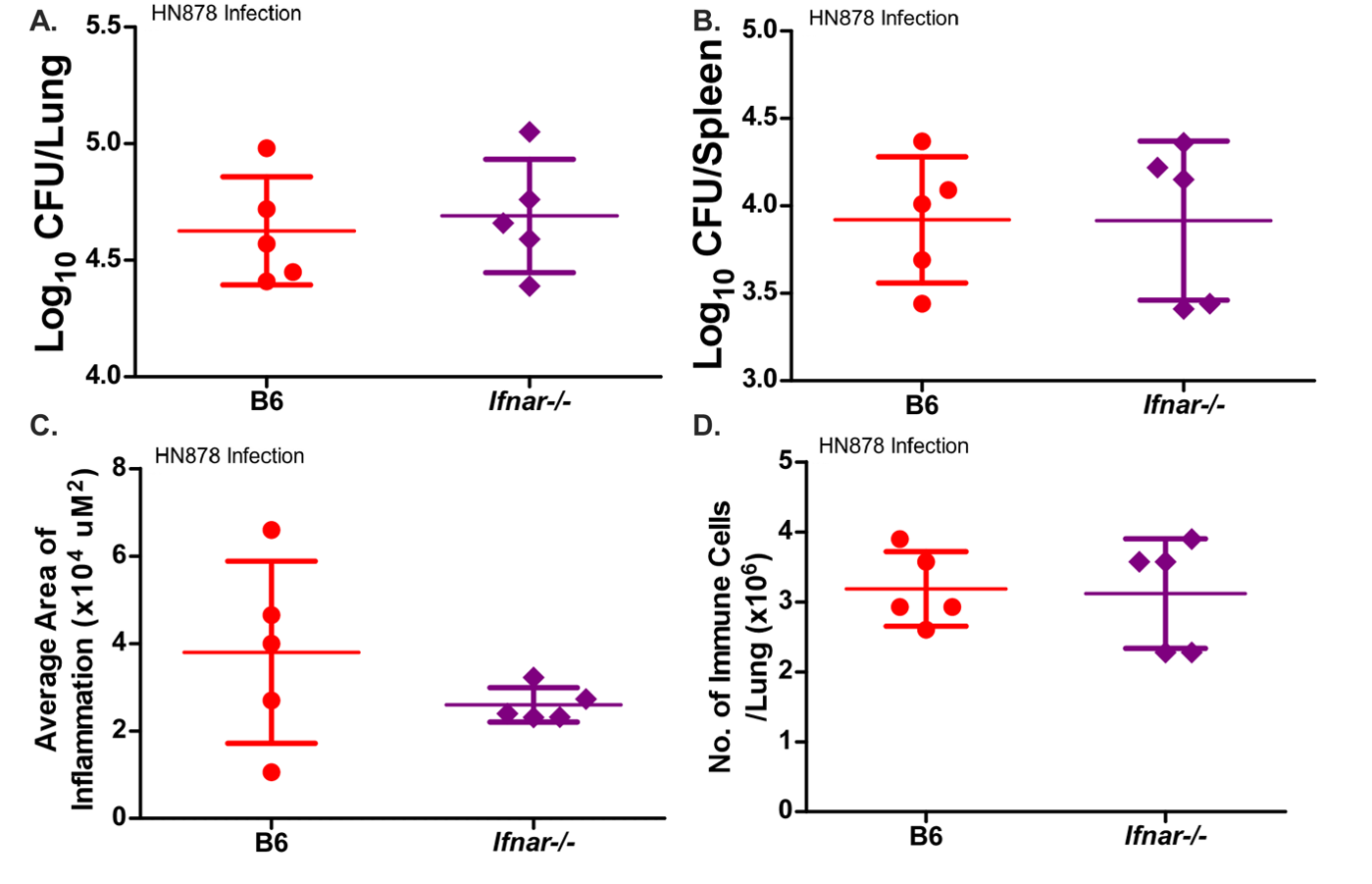

Supplement: Fig. S6 — Absence of type I IFN signaling does not impact wt HN878 Mtb infection. [file mbio.00946-23-s0006.png]
