# Supplementary material for: Mycobacterium tuberculosis carrying the rifampicin drug-resistance-conferring rpoB mutation H445Y is associated with suppressed immunity through type I interferons
Source: mBio. 2023 Sep 8;14(5):e00946-23. doi: 10.1128/mbio.00946-23 (PMC10653897; doi:10.1128/mbio.00946-23)
Supplement: Supplemental Figure Legends — Legends for all supplemental figures. [file mbio.00946-23-s0007.docx]

**Supplementary Figure 1**. ***Mycobacterium tuberculosis* (*Mtb*) isolates containing *rpoB*-H445Y SNP drive altered type I interferon production during infection.** A) Diagram depicting the presence or absence of SNPs in independent Mtb isolates. Briefly, drug-sensitive HN878 Mtb was cultured and plated on rifampicin-containing media. Spontaneous mutants were isolated, stocked, and fully sequenced. Isolates are grouped by their *rpoB* mutation and the presence of non-synonymous SNPs in each isolate is indicated with a circle in the respective column. B) C57Bl/6 bone marrow-derived macrophages were generated and infected with a multiplicity of infection of 1 with the indicated Mtb strains. Interferon-β protein levels in supernatants were quantified following 6 days of infection.

**Supplementary Figure 2. *rpoB*-H445Y *Mtb* infection limits myeloid cell recruitment and activation in mice.** B6 mice were aerosol infected with a low dose of *Wt* or *rpoB*-H445Y. The numbers of lung **(A)** neutrophils, **(B)** recruited macrophages (RMs), and **(C)** monocytes were determined by flow cytometry on 14 and 30 dpi. The numbers of **(D)**MHCII^+^ RMs and **(E)**monocytes were also determined. The data shown represent the means + SD of four to five biological replicates per experiment. Significant differences are indicated with asterisks (*, P < 0.05; **, P < 0.01) by Kruskal-Wallis test with Dunn’s multiple comparisons tests (A-D).

**Supplementary Figure 3. *rpoB*-H445Y *Mtb* infection induces altered cytokine and chemokine production in mice.** B6 mice were aerosol infected with a low dose of either *Wt* or *rpoB*-H445Y *Mtb*. **(A-E)** Cytokine and chemokine protein levels in lung homogenates were measured by ELISA and Lincoplex. The data shown represent the means + SD of four to seven biological replicates per experiment. Significant differences are indicated with asterisks (*, P < 0.05; **, P < 0.01; ***, P < 0.001) by Kruskal-Wallis test with Dunn’s multiple comparisons tests (A-E). One of two independent experiments shown.

**Supplementary Figure 4. *rpoB*-H445Y *Mtb* infection also results in a limited immune response in FeJ mice.** FeJ mice were aerosol infected with a low dose of *Wt* or *rpoB*-H445Y *Mtb*. **(A)**Formalin-fixed, paraffin embedded (FFPE) lung sections were H&E stained and the inflammatory area was measured. **(B)** Cytokine protein levels in lung homogenates were measured by ELISA. **(C)**Total number of mDCs and **(D)** MHC Class II^+^mDCs were determined by flow cytometry. Uninfected mice were included as controls (n=3). **(E)** The numbers of CD44^hi^ IFN-γ^+^ CD4^+^ T cells were also determined by flow cytometry following *ex vivo* stimulation of single cell lung suspensions with PMA, ionomycin, and Golgistop. The data shown represent the means + SD of three to six biological replicates per experiment. Significant differences are indicated with asterisks (*, P < 0.05; **, P < 0.01, ***, P < 0.001) by Kruskal-Wallis test with Dunn’s multiple comparisons tests (A-E). One of two independent experiments shown.

**Supplementary Figure 5. *Ifnar*^-/-^ mice have significantly enhanced myeloid cell recruitment and activation in the lung only after infection with *rpoB*-H445Y *Mtb*.**B6 and *Ifnar*^-/-^ mice were aerosol infected with a low dose of either *Wt* or *rpoB*-H445Y*Mtb* as indicated. Mice were sacrificed for analyses on 30 dpi. The number of lung **(A)** RMs and **(B)**monocytes were determined by flow cytometry. The numbers of MHC class II^+^**(C)**RMs and **(D)** monocytes were also determined by flow cytometry. The data shown represent the means + SD of four to five biological samples per experiment. Significant differences are indicated with asterisks (*, P < 0.05; **, P < 0.01) by the Mann Whitney test (A-D). One of two independent experiments shown.

**Supplementary Figure 6. Absence of type I IFN signaling does not impact *wt* HN878 *Mtb* infection.** B6 and *Ifnar*^-/-^ mice were aerosol infected with a low dose of *wt* HN878 *Mtb*. Mice were sacrificed at 30 dpi for subsequent analyses. Bacterial burden in the **(A)** lung and **(B)** spleen were determined by plating. **(C)**Formalin-fixed, paraffin embedded (FFPE) lung sections were H&E stained and the inflammatory area was measured. **(D)** Total numbers of lung immune cells in single cell suspensions were determined. The data shown represent the means ± SD of five to ten biological replicates per experiment. No significant differences were found by the Mann Whitney test (A-D). One of two independent experiments shown.
